# Melodic Intonation Therapy on Non-fluent Aphasia After Stroke: A Systematic Review and Analysis on Clinical Trials

**DOI:** 10.3389/fnins.2021.753356

**Published:** 2022-01-27

**Authors:** Xiaoying Zhang, Jianjun Li, Yi Du

**Affiliations:** ^1^School of Rehabilitation Medicine, Capital Medical University, Beijing, China; ^2^Beijing Key Laboratory of Neural Injury and Rehabilitation, China Rehabilitation Research Center, Beijing, China; ^3^Center of Neural Injury and Repair, Beijing Institute for Brain Disorders, Beijing, China; ^4^Department of Psychology, Music Therapy Center, China Rehabilitation Research Center, Beijing, China; ^5^Chinese Institute of Rehabilitation Science, Beijing, China; ^6^Key Laboratory of Behavioral Science, Institute of Psychology, Chinese Academy of Sciences (CAS), Beijing, China; ^7^Departments of Psychology, University of Chinese Academy of Sciences, Beijing, China

**Keywords:** melodic intonation therapy, music therapy, non-fluent aphasia, speech therapy, stroke

## Abstract

Melodic intonation therapy (MIT) is a melodic musical training method that could be combined with language rehabilitation. However, some of the existing literature focuses on theoretical mechanism research, while others only focus on clinical behavioral evidence. Few clinical experimental studies can combine the two for behavioral and mechanism analysis. This review aimed at systematizing recent results from studies that have delved explicitly into the MIT effect on non-fluent aphasia by their study design properties, summarizing the findings, and identifying knowledge gaps for future work. MIT clinical trials and case studies were retrieved and teased out the results to explore the validity and relevance of these results. These studies focused on MIT intervention for patients with non-fluent aphasia in stroke recovery period. After retrieving 128 MIT-related articles, 39 valid RCT studies and case reports were provided for analysis. Our summary shows that behavioral measurements at MIT are excessive and provide insufficient evidence of MRI imaging structure. This proves that MIT still needs many MRI studies to determine its clinical evidence and intervention targets. The strengthening of large-scale clinical evidence of imaging observations will result in the clear neural circuit prompts and prediction models proposed for the MIT treatment and its prognosis.

## Introduction

Aphasia is a language disorder generally caused by stroke-related damage to the dominant hemisphere. It describes a multitude of acquired language impairment as a consequence of brain damage (Go et al., 2014; WHO, 2015; Benjamin et al., 2017; Koleck et al., 2017). In relation to localization, it is possible to make a division between fluent and non-fluent aphasia. Oral expression of non-fluent aphasia is characterized by low speech volume, lack of grammar, and pronunciation dysphonia. According to the American Hearing Language Association's classification of aphasia, the types of non-fluent aphasia include motor aphasia, complete aphasia, transcortical motor aphasia, and transcortical mixed aphasia (Kim et al., 2016; Gerstenecker and Lazar, 2019; Hoover, 2019). According to the survey data from WHO on stroke prevention in 2019, about 2.6 to 4.7 million people suffer from stroke-related aphasia yearly, significantly impacting their quality of life (WHO, 2015; Wang et al., 2019). Aphasia affects the patient's linguistic skills and daily communication. As the course of the disease is prolonged, it will also impede patients' quality of life.

Due to the lack of targeted surgery and efficacious treatment regimens, speech therapy is a general method to train patients with aphasia. The mechanism of speech therapy is mainly based on the language function centers located in the dominant left hemisphere (Kamath et al., 2019). Several studies have demonstrated that music therapy for non-fluent aphasia is used to treat patients who have lost their speaking ability after a stroke or accident. It is reported that the right hemispheric regions are more active during singing (Jeffries et al., 2003; Callan et al., 2006; Ozdemir et al., 2006). Music therapy involving melodic elements is deemed to be a potential treatment for non-fluent aphasia, as singing potentially activates patients' right hemisphere to compensate for their lesioned left hemisphere (Zipse et al., 2009; Schlaug et al., 2010). Aside from singing, many other music therapy techniques have also been attempted, and the effectiveness of some methods has been revealed.

Melodic intonation therapy (MIT) is one of the verified effective methods of aphasia by the American Academy of Neurology (AAN) (Helm-Estabrooks and Albert, 2004). MIT is an intonation-based treatment method for non-fluent or dysfluent aphasic patients developed in response to the observation that severely aphasic patients can often produce well-articulated, linguistically accurate words while singing but not during a speech (Albert et al., 1973; Sparks et al., 1974). MIT is a hierarchically structured treatment that utilizes intoned (sung) patterns that exaggerate the typical melodic content of speech across three levels of increasing difficulty. At the elementary level, patients need to complete 1–2 syllables of melodic intonation in oral expression, such as “hello,” “thank you,” “goodbye,” etc. At the intermediate level, patients need to complete oral expressions of melodic intonation of 3–5 syllables, such as “I love you,” “I am thirsty (hungry),” “I have to rest,” etc. to express daily needs. At the advanced level, patients need to express sentences of 6–10 words or more, such as “I am going to train today,” “It is 10 a.m. in the morning,” etc. The original explanation of MIT is to utilize the musical and language output region in the right hemisphere, in which the mechanism differs from the left hemisphere (Albert et al., 1973; Sparks et al., 1974). An assumption raised by Albert and Sparks is that music can be effective by discovering music to language connections between the right and the left hemisphere in an interactive way or by using either reserved music/language functional area in the two hemispheres to speak. MIT combines melodic and rhythmic aspects of sentence intonation with language (Albert et al., 1973; Sparks et al., 1974; Sparks and Holland, 1976; Helm-Estabrooks et al., 1989; Cohen and Masse, 1993; Boucher et al., 2001; Norton et al., 2009). It can mobilize the auditory musical area on the right and the language area in the left hemisphere. The goal of MIT is namely to elicit the sound of the language (or spontaneous speech) by exaggerating the melody and rhythm of the language. The implementation process of MIT is musical, activating the right hemisphere mechanism that is not commonly used in daily language expression.

However, according to the currently published MIT studies, there is an excessive number of reviews and mechanism analysis studies. Still, there is a scarce number of randomized controlled trials (RCT), cross-over studies, cohort studies, and case studies. In experimental researches, the evidence is accentuated over language behavior measurements, and there are very few studies that use multimodal imaging observation to verify behavioral, neural mechanisms. In the assessment results of the language scale, the brain areas observed by MRI imaging are scattered, and the target areas of symptoms remain unclear. According to the results of existing mechanism analysis and scale evaluation, there are many possible narratives for the mechanism of MIT, but its underlying mechanism remains unclear as of yet (Breier et al., 2010; van de Sandt-Koenderman et al., 2010; Merrett et al., 2014; Zumbansen et al., 2014b). Therefore, the purpose of our review is to (1) retrieve the evidence and effectiveness of MIT for non-fluent aphasia after strokes, determine the superior performance of melody intonation therapy-related interventions in behavioral measurement results, and summarize our findings. (2) From a meager amount of MRI evidence, determine which areas the onset mechanism is more focused in, identify more targeted brain areas and circuits, and find a more feasible mechanism direction for the treatment of aphasia by MIT, thus providing the groundwork for future research.

## Materials and Methods

### Selection of Studies

We have planned and analyzed literature from reviews, systematic reviews, randomized controlled trials (RCT), clinical-controlled trials (CCT), cross-over studies, cohort studies, self-control, and case studies, regarding aphasia and music therapy. A literature search was conducted on four electronic databases: PubMed, Bing Scholar, Google Scholar, and Medline. The included articles are in English, French, Italian, Spanish, German, Korean, and Japanese. The publication timeframe was from January 1970 to July 2021. The keywords of “stroke,” “aphasia,” “music,” “melody,” “rhythmic,” “intonation,” “melodic intonation therapy,” “music therapy,” “music and aphasia,” and “rhythm and aphasia” were searched. The search was free and followed PRISMA's recommendations (Liberati et al., 2009; Higgins and Green, 2011), with a reference list of articles attached.

Randomized controlled trials (RCT), clinical-controlled trials (CCT), cross-over design, self-control, and case studies were subsequently recruited, with the omission of reviews. In accordance with the PICOS principle in evidence-based medicine, this review defines the criteria for inclusion.

(1) *Participants*: In participant's inclusion, all studies concerned only human adults (≥18 years) in stroke recovery period with non-fluent aphasia, including ischemic and hemorrhagic stroke, and the time since stroke was more than 2 weeks. (2) *Intervention*: The intervention group followed musical supported MIT such as melodic intonation therapy (MIT), modified MIT, rhythmic syllables therapy (RST), spoken language stimuli, singing therapy (ST), rhythmic therapy (RT), prosody perception task (PPT), sung-spoken story recall task, melodic cueing, melodic singing, and rhythmic cueing. (3) *Comparison:* The MIT intervention dose ranges from 1 to 4 times per week, and the duration ranges from 1 to 12 weeks. The control group was followed by speech therapy or blank control in the same dose and duration. (4) *Outcomes*: Using behavioral evaluation scales and fMRI to evaluate the results, the primary outcomes with a *p* < 0.5 are meaningful. (5) *Study design*: Methods are a randomized controlled study of MIT and speech therapy, or a self-controlled study of MIT, or a cross-design study of modified MIT and speech therapy, case reports of MIT, etc.

We compared speech therapy and melodic intonation therapy, combined with commonly integrated rehabilitation, and evaluated clinical outcomes.

### Data Sources and Search Strategy

**Figure 1:**
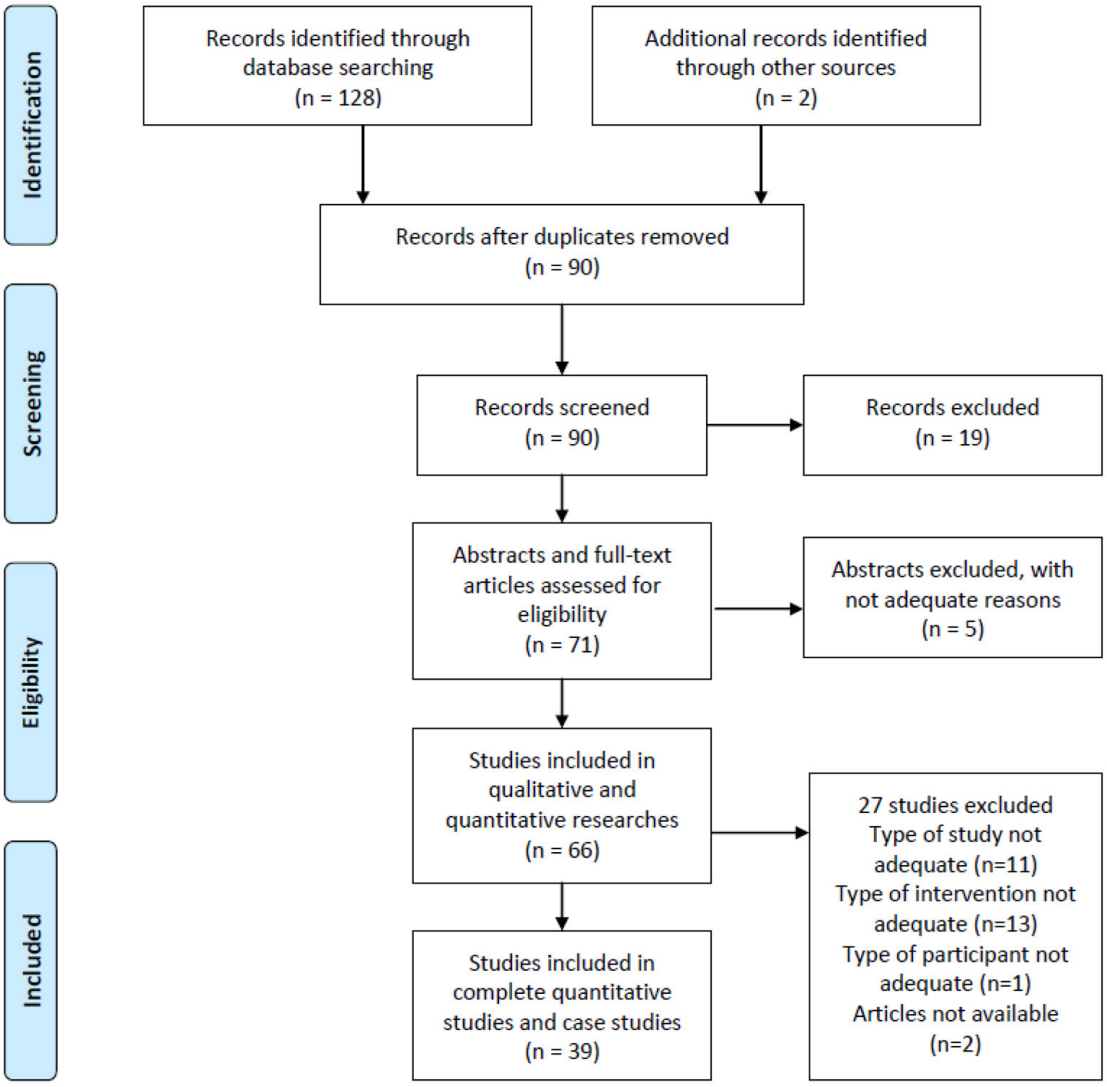
Flow diagram of article identification and inclusion.

After searching for relevant literature in 4 databases, a total of 128 works of literature about melodic intonation (induced) therapy were retrieved, and 2 was from another website. It was found that 90 articles were repeated in each database after reviewing the titles, indicating high reliability. After a quick review of the literature, 10 MIT literature reviews, 5 abstracts, and 4 qualitative analyses were excluded. The remaining 71 articles contain complete quantitative analysis and case studies. After careful examination of these articles, it was found that the data of 5 brief articles were published as spotlight, and 11 papers were presented as the original form; without statistical analysis, the statistical correlation could not be obtained. Thirteen articles did not belong to melodic intonation therapy and relative therapy. Three papers were for patients with a cognitive impairment not relevant to aphasia intervention. Finally, 39 quantitative experimental types of research and case studies of aphasia rehabilitation of typical MIT were identified. The risk of bias assessment was based on the handbook of Cochrane review methods (Higgins and Green, 2011; Figure 1).

The principle behind selecting these clinical studies as a systematic review is that these studies have applied MIT to clinical patients to observe the actual effects. Secondly, internationally standard measures were performed before and after the clinical trial to compare the results. Eleven of the experiments were accompanied by imaging tests. The intervention was a melodic musical form, accomplished by the therapist. Therefore, the above three points align with the therapy standards and principles proposed at the beginning of standard melodic intonation therapy.

## Main Results of Clinical Trials of MIT to Aphasia

This review summarizes all MIT studies with non-fluent aphasia patients since 1970 (Table 1). Since MIT was established in the 1970s as a more effective supplementary treatment for non-fluency aphasia, clinical trials on MIT have gradually garnered widespread attention. MIT clinical trials have the following characteristics: (1) In the research before the Twentieth century, the behavioral observation records of patients with MIT were more detailed; (2) Comparative case studies, self-controlled studies, and small sample experiments were more numerous; (3) Most of them used subjective language assessment scales for result evaluation. After the Twentieth century, with the advancement of imaging medicine, researchers conducted large sample experiments while focusing on behavioral measurements. They were more concerned about the evidence yielded by the brain imaging structure. The assessment tool was taken as a classification feature. Twenty-two MIT clinical trials evaluated using language ability scales and 11 clinical trials using imaging measurements; all the 33 pieces of research are listed in Table 1.

**Table 1:** The clinical trials of MIT for the aphasia of stroke.

**Trial**	**Design**	**Sample**	**Participants**	**Music therapist involved**	**Blinding**	**Intervention**	**Duration and dose**	**Primary outcome**	**Measures**	**Main results**
**Researches measured by scales (26)**										
Sparks et al. (1974)	Self-control	*n* = 8	8 Patients with severely impaired verbal output; good auditory comprehension; global aphasics; No improvement in verbal output for at least 6 months	No	No	Melodic intonation therapy (MIT) Programme	Daily therapy 3 months	Significant results	Boston Diagnostic Aphasia Examination (BDAE) (Goodglass and Kaplan, 1972)	Responsive naming–*F* = 25.3, df 1, 5; *P* = 0.005 Confrontation naming–*F* = 7.9, df 1, 5; *P* = 0.038 Phrase length–*F* = 29.6, df 1, 5; *P* = 0.003 Auditory commands–*F* = 2.2, df 1, 5; *P* = 0.198 Complex auditory material–*F* = 0.8, df 1, 5; *P* = 0.396 Reading comprehension, sentences-paragraphs–*F* = 0.3, df 1, 5; *P* = 0.50
Marshall and Holtzapple (1976)	Case study	*n* = 4	Patient 1: male; age: 49-years-time since stroke: 9 months Patient 2: male; age: 49-years-old time since injury: 25 months Patient 3: male; age: 53-years-old time since injury: 1 months Patient 4: male; age: 41-years-old time since injury: 1 months dignosis of left middle cerebral artery thrombosis with aphasia and severe apraxia.	No	No	Melodic intonation therapy (MIT) Modified melodic intonation therapy (MMIT)	60 min/day 3 days/week 3 months no return to normal speech	Significant results	Porch Index of Communication Ability (PICA) Overall Communicative Ability (OCA score)	Patient 1 has increased in PICA scores at 3 and 6 months post-MIT, and improved articulatory skills Patient 2 shows an increase of 8 scores (34–42) on the PICA Patient 3 and Patient 4 showed an improvement in verbal modality after MMIT
Goldfarb and Bader (1979)	Case study	*n* = 1	1 patient, male, 50-year-old with severe global aphasia after C for 10 years (left frontal thrombosis)	No	No	Melodic intonation therapy (MIT)	1 h/session 6 sessions/week 1 time in hospital 5 times at home	Significant results	Boston diagnostic aphasia examination (BDAE)	78% correct in speech mode; 84% correct in intonation without tapping mode; 92% correct in tonation plus tapping mode; and 88% correct without any hints
Popovic and Boniver (1992)	Self-control	*n* = 80	80 patient diagnosis with Broca aphasics and bucco-lingual apraxi	No	No	Melodic intonation therapy (MIT) in Romanian	60–120 min/session 7 sessions/week 2–4 weeks	Significant results	Romania aphasia test (RST)	MIT was considered an efficient method in the early stages of Broca aphasia with bucco-lingual apraxia
Springer et al. (1993)	Cross-over	*n* = 12	Group 1 (L/S Group) *n* = age: 46.17 ± 12.84 years time since stroke: 14.17 ± 14.11 months Group 2 (S/L Group) *n* = 6 age: 41.83 ± 9.72 years time since stroke: 26.5 ± 22.75 months	No	No	Melodic intonation therapy (MIT): MIT's facilitation technique used in a different therapeutic program MIT: linguistically oriented approach (L) MIT: stimulation approach (S)	60 min/day 3–4 days/week 2 weeks	Significant results	Aachen aphasia test (AAT) Token test (TT) Communicative abilities in daily living (CADL) Amsterdam-Nijmegen everyday language test (ANELT)	There's significantly improvement for tasks with temporal items with spatial items (*p* = 0.042) and wh-words (*p* = 0.071); there's no significant differences in written multiple-choice task (*p* = 0.583) Non-parametric tests had significantly larger direct effects for the linguistically oriented learning approach (L), but significantly larger post-effect for the stimulation approach (S)
Baker (2000)	Case study	*n* = 2	Patient 1: female 32-year-old, trauma brain injury in left hemisphere time since injury: 9 months Patient 2: male, 30-years old, trauma brain injury in left hemisphere time since injury: 4 months	Yes	No	Modified melodic intonation therapy (MMIT): Specific musical line and accompaniment for each trained sentence	30 min/session 3–8 sessions/week 4–27 months	Significant results	Functional language of 180 words/phrases Functional language of 45 words	Patient 1 had acquired a functional language of 148 words/phrases Patient 2 was able to independently generate the 30 words
Bonakdarpour et al. (2003)	Self-control	*n* = 7	7 patients with non-fluent aphasia Mean age: 52.4 years (45–61 years) time since stroke: 35.43 months (14–58 months)	No	No	Melodic intonation therapy in Persian (MIT-P): Exaggeration of normal prosody	3–4 days/week 1 month	Significant results	Wilcoxon signed-rank test Farsi aphasia test (FAT) Brain CT scan for diagnosis	Wilcoxon signed rank test showed statistically significant improvement in phrase length (*p* = 0.0125); number of correct content units (*p* = 0.0107); confrontational naming (*p* = 0.0312); responsive naming (*p* = 0.0107); repetition (*p* = 0.0084); word discrimination (*p* = 0.238); commands (*p* = 0.238)
Wilson et al. (2006)	Case study	*n* = 1	1 patient, male, 48-years-old with a left middle cerebral artery tertiary stroke for 4 years	Yes	No	3 methods of trained items Method 1: Melodic intonation therapy (MIT) Method 2: Repetition training Method 3: Unrehearsed training	2 days/week 1 month	Significant results	Magnetic resonance imaging (MRI) for diagnosis Boston diagnostic aphasic examination (BDAE) Australian music examinations board (AMEB)	Compared to Method 2 and Method 3, Method 1 showed a significant main immediate effect of time, *F*_(1, 56)_ = 6.47, *p* < 0.05, and phrase group, *F*_(2, 56)_ = 13.9, *p* < 0.001, and a significant interaction between time and group, *F*_(2, 56)_ = 9.95, *p* < 0.001. The results showed a significant long time effect of phrase group, *F*_(1, 37)_ = 5.08, *p* < 0.05, and a significant interaction effect between time and group, *F*_(1, 37)_ = 5.4, *p* < 0.05
Racette (2006)	Self-control	*n* = 8	4 Severe Broca's aphasia 4 Moderate to severe mixed aphasia age: 36–67 years (mean 51.63 years) (mean 8 years) time since stroke: 5–19 years	No	No	Experiment 1: Aphasic patients repeated and recalled familiar songs Experiment 2: Aphasic patients repeated and recalled lyrics from novel songs Experiment 3: With an auditory model while learning novel songs, aphasics repeated and recalled more words when singing than when speaking	Once experimental time, three times		Neuro- psychological battery of tests Standard non-colored Raven's matrices Tower of London Montrea battery for evaluation of amusia (MBEA)	Singing perse does not help aphasics to improve their speech, whether the songs were familiar (Experiment 1) or unfamiliar (Experiment 2) But in Experiment 3, with an auditory model while learning novel songs, aphasics is better than speaking and singing in experiment 1 and 2
Kim and Tomaino (2008)	Self-control	*n* = 7	5 Severe Non-fluent aphasia 2 Moderate aphasia time since stroke: 14.28 years	Yes	No	Music therapist supported music therapy Familiar songs singing Breathing into single-syllable sounds Musically assisted speech Rhythmic speech cueing, Vocal intonation Dynamically cued singing Oral motor exercises	30 min/session 3 times/weeks 4 weeks		Neuro- psychological battery of tests Video measurement	Speech and singing carefully enhance each patient's expectancy in achieving improved performance of word retrieval, prosody and articulation
Hough (2010)	Case study	*n* = 1	1 patient, male, 69-years-old with chronic Broca's aphasia after left cerebro-vascular accident of 4 years' duration	No	No	Modified melodic intonation therapy (MMIT)	3h sessions/week 8 weeks Follow-up at 2–4 weeks	Significant results	Western aphasia battery-revised (WAB-R); Aphasia quotient (AQ); Cortical quotient (CQ); American speech-language hearing Association functional assessment of communication skills (ASHA FACS)	After MIT, the patient reached 75% accuracy on automatic phrases at 4 weeks; self-generated phrases was 55% at 8 weeks The results revealed a significant difference in the automatic phrase data between baseline and post-treatment data (*t* = 18.7314; df = 6.456; *p* < 0.00001) The results revealed a significant difference in the self-generated phrase data between baseline and post-treatment data (*t* = 33.3729; df = 10; *p* < 0.00001) AQ increased 13 scores and CQ increased 13.6 after MIT
Vines et al. (2011)	Self-control	*n* = 6	Median age: 30–81 years time since stroke: at least 1 year	No	No	Melodic intonation therapy (MIT) transcranial direct current stimulation (tDCS)	EG: 20 min/day CG: 20 min/day 3 days	Significant results	Boston diagnostic aphasia Examination(BDAE)	Applying anodal-tDCS during MIT produced a significantly greater improvement in verbal fluency
Van der Meulen et al. (2012)	Clinical trial no. NTR 1961	*n* = 2	Patient 1 age: 29 years time since stroke: 8 months Patient 2 age: 25 years time since stroke: 2 weeks	No	No	EG: melodic intonation therapy (MIT) CG: No language therapy	3–5 h/week 6 weeks	Significant results	Aachen aphasia test (AAT) Amsterdam nijmegen everyday Language test (ANELT) Sabadell story retell task	Patient 1 improved 5 scores in repetition and 5 scores in comprehension (AAT) Patient 2 improved 35 scores in repetition trained phrases; 50' in repetition; 7' in action naming and 9' in comprehension (AAT). 7 scores was improved in ANELT; 22.5 scores was improved in Sabadell
Conklyn et al. (2012)	RCT pilot	*n* = 30	EG *n* = 10, age: 56.8 ± 17.11 years time since stroke: 32.2 ± 93.42 days CG *n* = 14, age: 66.9 ± 11.77 years time since stroke: 28.4 ± 67.84 days	Yes	Single	EG: modified melodic intonation therapy (MMIT) CG: Music therapist discussed with the patient	15 min/session 3 sessions	Significant results	Western aphasia battery (WAB)	Compared to the control, MIT group adjusted total items 1–3 score (*p* = 0.02); 2–3 score (*p* = 0.02) and responsive items 2–3 score (*p* = 0.02)
Stahl et al. (2013)	Self-control Cross-over Cross-over	*n* = 3	Median age: 56.2 years time since stroke: 23.47 months	No	No	Formulaic singing therapy (MT) Rhythmic therapy (RT) Standard therapy (ST)	1 h/session 3sessions/week 6 weeks	Significant results	Aachen aphasia test (AAT) Sabadell story retell task	Compare to RT and ST, MT group improved significantly (*p* = 0.001) in repetition; MT group improved in spontaneous words and is stable after 3 months.
Lim et al. (2013)	CCT	*n* = 21	EG *n* = 12, age: 56.5 years time since stroke: 187 days CG *n* = 9, age: 62.7 years time since stroke: 2,473 days	No	No	EG: melodic intonation therapy (MIT) CG: speech language therapy (SLT)	2 × 60 min/week 4 weeks	Significant results	Western Aphasia Battery in Korean version (K-WAB) Aphasia quotient (AQ)	In chronic group, MIT improved AQ (*p* = 0.126); spontaneous speech (*p* = 0.126); comprehension (*p* = 0.429) and repetition (*p* = 0.177) In subacute group, MIT improved AQ (*p* = 0.476); comprehension (*p* = 0.067) and naming (*p* = 0.352)
Zumbansen et al. (2014a)	Self-control Cross-over	*n* = 3	Median age: 51.67 years time since stroke: 21.67 months	No	No	Melodic intonation therapy (MT) Rhythmic syllables therapy (RT) Spoken syllables therapy (ST) Patient 1: MT-RT-ST Patient 2: RT-ST-MT Patient 3: ST-MT-RT	1 h/session 3 sessions/week 6 weeks	Significant results in MIT	MT-86 aphasia battery Verbal fluency test Montreal battery of evaluation of musical abilities (MBEMA) Wechsler Adult intelligence scale–III (WAIS-III); Wechsler memory scale-III (WMS-III); Computer syllables tests (cordial analyseur)	Compare to RT and ST, patient 1 improved significantly (*Z* = −2.101, *p* = 0.036) in MIT; patient 2 improved in MIT (*Z* = −2.017, *p* = 0.044); patient 3 improved in MIT (*Z* = −2.329, *p* = 0.024)
Van der Meulen et al. (2014)	RCT Cross-over	*n* = 27	EG *n* = 16, age: 53.1 ± 12.0 years time since stroke: 9.3 ± 2.0 weeks CG *n* = 11, age: 52.0 ± 6.6 years time since stroke: 11.9 ± 5.9 weeks	No	No	EG: melodic intonation therapy (MIT) CG: followed by delayed MIT	5 h/week 6 weeks	Significant results	Aachen aphasia test (AAT) Amsterdam Nijmegen everyday language test (ANELT) Semantic association task (SAT) Sabadell story retell task MIT repetition	Compared to the control group, MIT improved Repetition (AAT) (*p* = 0.05); MIT-repetition (*p* < 0.01); trained items (*p* < 0.01); untrained items (*p* = 0.25) There is no significant difference in Sabadell (*p* = 0.82); ANELT (*p* = 0.07) and Naming (AAT) (*p* = 0.10)
Cortese et al. (2015)	Self-control	*n* = 6	Median age: 59.8 ± 9.3 years Range: 53–71 years time since stroke: 9 months	No	No	Melodic-rhythmic therapy in Italian	30–40 min/day 4 days/week 16 weeks	Significant results	Aachen aphasia test (AAT)	In Italian MRT, phonemic structure, speech automatism, prosody, communication, correct repetition, naming and comprehension improved (*p* = 0.031)
Raglio et al. (2015)	RCT	*n* = 30	EG *n* = 10, CG *n* = 10 chronic aphasia	No	No	EG: singing active music therapy CG: speech language therapy	30 min/day 15 weeks	Significant results	Aachen aphasia test (AAT) Short form health survey	The study shows a significant improvement in spontaneous speech in the experimental group (Aachener Aphasie subtest: *p* = 0.020; Cohen's *d* = 0.35); the 50% of the experimental group showed also an improvement in vitality scores of short form health survey (chi squared 4.114; *p* = 0.043)
Van Der Meulen et al. (2016)	RCT Cross-over	*n* = 17	EG *n* = 10, age: 58.1 ± 15.2 years time since stroke: 33.1 ± 19.4 months	No	No	EG: melodic intonation therapy (MIT) CG: no language therapy	EG: 5 h/week 1–6 weeks	Significant results	Aachen aphasia test (AAT) Amsterdam-Nijmegen everyday	1–6 weeks: compare to CG, EG (MIT) group improved in trained items (*p* = 0.02); untrained items (*p* = 0.40)
			CG *n* = 10, age: 63.6 ± 12.7 years time since stroke: 42.6 ± 23.7 months				CG: 5 h/week 7–12 weeks		Language test (ANELT) Correct information units (CIU) Semantic association test (SAT)	7–12 weeks: compare to CG, EG (MIT) group improved in trained items (*p* < 0.01); untrained items (*p* < 0.01)
Slavin and Fabus (2018)	Case study	*n* = 1	Age: 63 years old Time since stroke: 10 years	Yes	No	Melodic intonation therapy (MIT)	50 min/session 2 sessions/week 12 weeks	Significant results	Boston diagnostic aphasi Examination (BDAE), Apraxia battery for adults II edition	MIT improved auditory comprehension skills, answering questions, and repetition of BDAE after listening to paragraphs
Martínez et al. (2018), Trial no. NCT3433495	Randomized cross-over pilot trial	n=20	EG *n* = 10, age: 66.05 ± 14.9 years time since stroke: 18.9 ± 13.43 months EG *n* = 10, age: 61.4 ± 13.7 years time since stroke: 24.1 ± 16.35 months	No	No	EG: Spanish adaptation of melodic intonation therapy (S-MIT) CG: delayed MIT	30 min/session 12 sessions 6 weeks	Significant results in CAL	Boston diagnostic aphasia examination (BDAE) Communicative activity log (CAL)	Compared to the control group, S-MIT improved communicative activity log (CAL) (*p* = 0.048) There is no significant difference in comprehension (*p* = 0.925) and repetition (*p* = 0.727) of BDAE between two groups
Kasdan and Kiran (2018)	Self-control	*n* = 40	EG *n* = 16, age: 61.25 ± 10.19 years CG *n* = 16, age: 63.0 ± 10.26 years time since stroke: not specified	No	No	Songs Melodic intonation therapy (MIT)	1 h/session	Significant results	Western aphasia battery (WAB Aphasia quotient (AQ)	MIT improved a three-factor repeated measures, phrase length (*p* < 0.001); a between-subjects effect of group (*p* < 0.001)
Leo et al. (2019)	RCT	n=31	EG *n* = 17, age: 54.4 ± 11.3 years time since stroke: 3 weeks−6 months CG *n* = 14, age: 51.4 ± 17.7 years time since stroke: 3 weeks−6 months	No	No	Music perception task Prosody perception task Sung-spoken story recall task	1–1.5 h/day 3 weeks	Significant results	NIHSS score BDAE aphasia severity rating scale MBEA scale and rhythm RBMT story recall immediate	In the two tasks, the aphasic patients recalling longer in the sung than spoken task (*p* = 0.013); emotional prosody perception correlated significantly with the recall in the sung task (*p* < 0.001); and also with chunk length in the
Zhang et al. (2021)	RCT	*n* = 40	EG *n* = 20; age: 52.90 ± 9.08 years CG *n* = 20; age: 54.05 ± 10.81 years	Yes	No	EG: melodic intonation therapy in Chinese CG: Speech therapy in Chinese	0.5 h/day 5 times/week 8 weeks	Significant results	Boston diagnostic aphasia examination (BDAE) Hamilton anxiety scale (HAMA) Hamilton depression scale (HAMD)	In the spontaneous speech (information, *p* = 0.0002), the listening comprehension (true or false, *p* = 0.0019; word recognition„ *p* = 0.0001; and sequential order, *p* = 0.0001), fluency (*p* = 0.0019), repetitions (*p* = 0.0019), and naming (*p* = 0.0001) of the intervention group were significantly higher than the control group in terms of the cumulative effect of time and the difference between groups after 8 weeks
**Researches measured by imaging (13)**										
Naeser and Helm-Estabrooks (1985)	Original	*n* = 8	Good response group (GR) *n* = 4 age: 49.5 ± 10.69 years Poor response group (PR) *n* = 4 age: 41.75 ± 14.91 years	No	No	Melodic intonation therapy (MIT)	Over 1–8weeks	Significant results	Boston diagnostic aphasia examination (BDAE) **CT scan**	GR cases improved in speech characteristics ratings for phrase length and grammatical form on the BDAE; the PR cases showed no improvement GR cases had lesions which involved Broca's area and white matter deep to it plus large superior lesion extension into peri ventricular white matter deep to the lower motor cortex area for face, and had no large lesion in Wernicke's area and no lesion in the temporal isthmus or the right hemisphere PR cases had bi-lateral lesions or lesion including Wernicke's area or the temporal isthmus
Laine et al. (1994)	Self-control pilot study	*n* = 3	Patient 1: Chronic global aphasia male, 58-years-old, 5 months after stroke Patient 2: chronic mixed non-fluent aphasia; male; 58-years-old; 16 months after stroke Patient 3: chronic Wernicke's aphasia; 62-years-old; male; 4 months after stroke	No	No	Repetition of words and sentences either with normal prosody or intoned (intoned vs. normal speech)	45 min/session 3 sessions/week 3.5 months	Significant results	Boston diagnostic aphasia examination (BDAE); **CT Scan single photon emission computed tomography (SPECT)**	Patient 1 showed a totally uniform pattern in the relative perfusion changes. His pattern indicated increased left hemisphere, not right hemisphere activation during MIT Patient 2 and 3 did not find evidence for increased right hemisphere activation during MIT
Belin et al. (1996)	Self-control pilot study	*n* = 7	2 Chronic Broca's aphasia; 5 chronic global aphasia age: 40–58 years (mean, 49.7 years) time since stroke: 4 to 41 months; (mean ± SD, 19 ± 15 months)	No	No	Repetition of sentences either with normal prosody or intoned (normal speech vs. silence; intoned vs. normal speech)	the duration of MIT in French therapy ranged from 37–42 months	Significant results	Boston diagnostic aphasia examination (BDAE); Wilcoxon signed-rank test **positron emission tomography (PET)**	Significantly more words (*p* < 0.03, Wilcoxon's rank sum test) were correctly repeated with MIT (16.3 ± 8 words) than without MIT (12.4 ± 8 words) Two findings: 1st, simple passive (word hearing) and active (word repetition) verbal tasks performed without MIT resulted in abnormal activation of right hemisphere structures, homotopic to those normally activated in the intact left hemisphere. 2nd, word repetition performed with MIT reactivated Broca's area and the adjacent left prefrontal cortex
Schlaug et al. (2008)	Case study randomly assigned	*n* = 2	2 patients with severe non-fluent aphasia as the result of a left hemisphere ischemic stroke Patient 1: male; age 47; time since stroke: 13 months Patient 2: male; age 58; time since stroke: 12 months	No	No	Melodic intonation therapy (MIT) Speech repetition therapy (SRT) Patient 1 MIT vs. Patient 2 SRT	90 min/day 5 days/week Over 8 weeks totally 70 sessions	Significant results	Correct information units (CIUs) Activities of daily living (ADL) Boston diagnostic aphasia examination (BDAE); **Functional magnetic resonance imaging (fMRI)**	MIT-treated patient had greater improvement on all outcomes than the SRT treated patient Patient 1 showed significant fMRI changes in a right-hemisphere network involving the premotor, inferior frontal, and temporal lobes Patient 2 had changes in a left hemisphere network consisting of the inferior pre- and post-central gyrus and the superior temporal gyrus
Schlaug et al. (2009)	Self-control pilot study	*n* = 6	Median age: not specified chronic aphasia patients time since stroke: at least 1 year	No	No	Melodic intonation therapy (MIT)	75 sessions	Significant results	**Magnetic resonance imaging (MRI); Diffusion tensor imaging (DTI)**	All six patients showed a significant increase in the absolute number of fibers in the right AF comparing post- vs. pre-treatment DTI studies (paired *t*-test, *p* = 0.04) and also an increase in the fiber length
Breier et al. (2010)	Case study	*n* = 2	Patient 1 age: 55 years old time since stroke: 5 years Patient 2 age: 49 years old time since stroke: 2 years	No	No Single	Melodic intonation therapy (MIT)	30 min/session 2 session/day 2 days/week 3 weeks	Controversial results	Action naming test **Magneto-** **encephalo-** **graphy (MEG)**	Patient 1 exhibited a significant increase in CIUs (>35%) after the first block of treatment. This improvement was maintained after the break Patient 1, who was improved in language function to MIT, exhibited a steady reduction in activation within the right hemisphere across the two therapy blocks, resulting in a strong left hemisphere lateralization of MEG activity Patient 2, who did not respond positively to MIT, exhibited increased right hemisphere activation after both blocks of therapy compared to baseline, resulting in a right hemisphere lateralization of MEG activity
van de Sandt-Koenderman et al. (2010)	Case study	*n* = 1	1 patient with Broca's aphasia in the subacute stage post-stroke Age: 25 years old; female time since stroke: 2 weeks	Not	No	Melodic intonation therapy (MIT)	1 h/session 5 sessions/week 2–8 weeks	Significant results	Aachen aphasia test (AAT): CIUs/minute Story retelling; **functional magnetic resonance imaging (fMRI)**	AAT: spontaneous speech 1/5–3/5; repetition *T* = 39–>*T* = 47; naming *T* = 39–>*T* = 46; CIUs/min 22.5–>55 fMRI: left more than right IFG, left superior and middle temporal gyrus and perilesional region in the angular/ supermariginal gyus, left caudate nucleus, bilateral supplementary, cingulate and premotor areas, left prefrontal cortex.
Zipse et al. (2012)	Case study	*n* = 1	1 patient with stroke resulted in very large left-hemisphere lesion age: 11-year-old	No	No	Melodic intonation therapy (MIT)	1.5 h/session 5 sessions/week 16 weeks 80 sessions 120 h totally	Significant results	**Functional magnetic resonance imaging (fMRI)** **Diffusion tensor imaging (DTI)**	fMRI: There was an increase in activation in right supplementary motor areas after 40 sessions and higher levels of activation in the right posterior middle temporal gyrus (MTG), occipital cortex, and possibly cerebellum. It showed a strong increase in activation of right posterior middle frontal and inferior frontal areas DTI: Both the arcuate fasciculus (AF) and uncinate fasciculus (UF) increased in volume at the beginning, midpoint and the conclusion
Al-Janabi et al. (2014)	Case study	*n* = 2	Patient 1 age: 65 years old time since stroke: 18 months Patient 2 age: 49 years old time since stroke: 20 months	No	No	Melodic intonation therapy (MIT) Excitatory repetitive transcranial magnetic stimulation (rTMS)	20 min/day 6 days	Significant results	Western aphasia battery (WAB) **Functional magnetic resonance** **imaging (fMRI); 3T MR system**	Patient 1 revealed significant activity increase in left BA44, *t* = 1.79, *p* < 0.05 and decrease in right BA44, *t* = 2.92, *p* < 0.01 Patient 2 revealed significant activity increase in left BA44, *t* = 1.77, *p* < 0.05, right BA44, *t* = 1.77, *p* < 0.05, left BA45, *t* = 3.51, *p* < 0.001
Jungblut et al. (2014)	Case study	*n* = 3	Patient 1 age: 53 years old time since stroke: 18 months Patient 2 age: 44 years old time since stroke: 18 months Patient 3 age: 44 years old time since stroke: 18 months	No	No	Melodic intonation therapy (MIT)	Once experimental time	Significant results	Hierarchical word list (HWL) **Functional magnetic resonance imaging (fMRI)**	pre and posttreatment assessments of patients' vocal rhythm production, language, and speech motor performance yielded significant improvements for all patients In the left superior temporal gyrus, whereas the reverse subtraction revealed either significant activation or right hemisphere activation
Orellana et al. (2014)	Same group self-control	*n* = 20	Median age: 43 years Range: 21–51 years time since stroke: not specific	No	No	Melodically intoned stimuli Spoken language stimuli	2 conditions were completed in 1 experiment 30 min totally	Significant results	Functional Magnetic resonance **imaging (fMRI); 3T MR system**	Compared to spoken items, melodic > spoken. For melodic intoned items, increased activation was seen left-lateralized in the SMG, IPL, middle and superior temporal gyrus, middle and superior frontal gyrus, Right-lateralized activation was seen in the insula, rolandic operculum, and pars opercularis of the inferior frontal gyrus
Akanuma et al. (2015)	Self-control	*n* = 10	Median age: 63.7 years Months from onset: 111.5	No	No	singing melodically therapy	30 min/session 1 session/week 10 weeks	Significant results	**Positron emission tomography (PET)**	5 patients exhibited improvements after singing intervention; all exhibited intact right basal ganglia and left temporal lobes
Tabei et al. (2016)	Case study	*n* = 1	1 Patient, age: 48 years old time since stroke: 3 years	Yes	No	Japanese version of melodic intonation therapy (MIT-J)	45 min/day 9 days	Significant results	Japanese version of western aphasia battery (J-WAB) Naming of 90 words aphasia quotient (AQ) Japanese version of Raven's Colored Progressive matrices (J-RCPM) Benton visual retention test (BVRT) **Functional MRI 3.0-T MR scanner**	After MIT, the patient improved 4 points in spontaneous speech; 0.9 in auditory comprehension; 0.8 in repetition; 1.3 in naming of J-WEB; 14 in AQ; 6 in correct naming words and 1.78 seconds in response time fMRI showed a significant activation of medial frontal gyrus, inferior frontal gyrus, superior temporal gyrus, lentiform nucleus, and lingual gyrus of the right hemisphere

*The experimental researches on MIT from 1970 to present. It is divided into two parts. The first part is the experimental researches that used scales to measure the results. The second part is the experimental researches that used imaging for verification. EG, experimentalgroup; CG, ControlGroup; RCT, randomisedcontrolled trial; DTI, diffusion tensor imaging; MR, magnetoencephalography; SMG, supramarginal gyrus; IPL, inferior parietal lobule; MEG, magnetoencephalogram; tDCS, transcranial direct-current stimulation; BA, brodmann area*.

### The Effects of MIT on 15–40 Sample Trials: The Most Assessment Tools Are Subjective Measurement Scales

In these MIT clinical trials, 13 experiments use various language assessment scales for evaluation, accounting for the majority. Melodic interventions are the selected essential factors, but evaluation criteria are equally important. There are mainly two evaluation criteria in the quantitative studies, one is various standard language test scales, which include the Boston Diagnostic Aphasia Examination (BDAE), the Western Aphasia Battery (WAB) in different language versions, the Aphasia Quotient (AQ), the Aachen Aphasia Test (AAT), amongst others. The other is imaging check, which includes functional magnetic resonance imaging (fMRI), magnetic resonance (MR), and diffusion tensor imaging (DTI), which are usually applied in a one-time assessment.

#### RCT Studies Evaluated Using Standard Language Test Scales Showed Consistent Results in Behavioral Measurement Results (Without Imaging)

Of the more than 15 MIT RCT studies selected in this review, seven valuable clinical trials used the language assessment scale to evaluate the results. Conklyn et al. (2012); Lim et al. (2013); Van der Meulen et al. (2014); Van Der Meulen et al. (2016); Raglio et al. (2015); Kasdan and Kiran (2018); Haro-Martínez et al. (2019); Leo et al. (2019), and Zhang et al. (2016, 2021) all used various language scales to assess two groups of patients with aphasia. The results demonstrated that whether it was only one observation of the immediate treatment effect or the cumulative treatment effect for up to 12 weeks, compared with the speech therapy group, the MIT group was better in understanding (Haro-Martínez et al., 2019), retelling (Haro-Martínez et al., 2019), and oral task response time (Lim et al., 2013), and oral memory time and retelling phrase length (Kasdan and Kiran, 2018) have been markedly improved. Regarding spontaneous expression, most of the target languages trained by MIT are short sentences of varying lengths, while the content of melody training is fixed. Therefore, in addition to improving the level of training items, patients receiving MIT can also enhance the spontaneous speech of untrained items. This is particularly conspicuous in the test of story retelling (Van der Meulen et al., 2014; Van Der Meulen et al., 2016). These meaningful behavioral measurement results are reflected in the scores of different dimensions of various language test scales.

Among the specific results, Haro-Martínez et al. (2019) found that after MIT, the MIT group improved communicative activity log (CAL), but no significant difference was noted in comprehension and repetition. Leo et al. (2019) found that after singing melody in the MIT group, the aphasic patients recalled longer in the singing rather than the speaking task and also with chunk length in the singing task. Kasdan and Kiran (2018) compared 1-h immediate effect after MIT and then found that patients with standard MIT conspicuously improved phrase length. Zumbansen et al. (2014a) conducted a crossover study on 3 aphasia patients for 6 weeks to compare MIT. The results showed that all of the 3 patients in MIT training improved clarity of syllables significantly. Stahl et al. (2013) did a similar crossover study of 3 aphasia patients, and it turns out the MT group improved significantly in repetition. In 2014, Van der Meulen et al. (2014) and Van Der Meulen et al. (2016), conducted an MIT crossover study on 27 aphasia patients, among which 16 patients received MIT for 6 weeks, and 11 patients in the control group received MIT after weeks. It was revealed that compared to the control group, the MIT group improved the repetition (AAT) in both trained items and untrained items. He then ran the same MIT crossover study in 2016 and found MIT group improved repetition in trained items and spontaneous sentences in untrained items. Raglio's et al. RCT study (Conklyn et al., 2012; Lim et al., 2013; Raglio et al., 2015) also proved that MIT improved repetition, listening comprehension, spontaneous speech, naming, and responsive items 2–3 score. Vian (Vines et al., 2011) turns out that applying anodal-tDCS during MIT produced a significantly greater improvement in verbal fluency.

#### Case Studies and Small Sample Studies Have the Characteristics of Complete Specific Treatment Interventions

There are 6 clinical trials with sample sizes between 1 and 6 patients. These studies mostly use the patient's control or crossover design to observe the effectiveness of MIT intervention. Due to the small sample size, these studies reflect the characteristics of a more detailed record of the intervention process and a more evident division of music elements in MIT. In the MIT intervention conducted by Van der Meulen et al. (2012) for 2 patients, a dedicated MIT therapist carried out the implementation process. Although in the MIT study conducted by Racette (2006), Kim and Tomaino (2008), Stahl et al. (2013), Zumbansen et al. (2014b), and Cortese et al. (2015), the implementer of the intervention process was realized by a speech therapist. Still, because the case study can record the detailed procedure, they compared the difference between melody and rhythm and found that the melody is dominant. The prognostic score will display more positive results. In the case report by Slavin and Fabus (2018), the therapist trained in NMT who performed MIT treatment also showed positive results. Although the samples in the above studies are generally small, the results are similar to the RCT study of more than 15 people, and the intervention process tends to be more musical.

#### The Advantages and Disadvantages of RCT Studies of Using Medical Imaging or Computers for Evaluation

In the clinical trials reviewed, most of the studies using MRI have the following characteristics: (i) case studies are dominant; (ii) the number of subjects is inferior or equal to 6; (iii) in case of large sample size, MRI observation should only be used before and once after MIT intervention to provide an immediate comparison. The above three characteristics are in an either-or relationship and will not appear simultaneously in the same study. In addition, we also found that the number of MIT musical interventions directly leads to different imaging results.

Among the RCT studies searched for, eight types of research used MRI to compare the effect before and after treatment. Orellana et al. (2014) compared an immediate impact on 20 aphasia patients before and after once MIT. After the intervention, fMRI and 3T MR scans showed that MIT increased activation in the left-lateralized in the SMG, IPL, STG, and SFG. Right-lateralized activation was seen in the insula, rolandic operculum, and pars opercularis of the inferior frontal gyrus. Akanuma et al. (2015) used positron emission tomography (PET) to conduct a self-control study in 10 chronic aphasia patients. The results demonstrated that 5 patients exhibited improvements after singing intervention; all indicated intact right basal ganglia and left temporal lobes. Norton et al. (2009), Schlaug et al. (2009), and Zipse et al. (2012) performed DTI to analyze structural changes in both hemispheres in 7 patients before and after MIT intervention. It turns out that all 7 patients showed a substantial increase in the absolute number of fibers in the right arcuate fasciculus (AF) comparing post-vs. pre-treatment DTI studies (paired *t*-test, *p* = 0.04) and also an increase in the fiber length, although omitting to mention the professional music therapists. It is worth noting that their melodic intervention time all exceeded 8 weeks, 75 courses of treatment. Al-Janabi et al. (2014) observed patients with functional magnetic resonance imaging after 6 days of MIT intervention and found that the left BA44 and right BA44 of the patients who received MIT had a significant increase in the activity. But Breier et al. (2010) compared two patients with chronic aphasia and came up with contradictory results. He showed a steady decrease in activation in the right hemisphere of both treatment areas, resulting in strong left hemisphere lateralization of MEG activity. However, Jungblut used his case studies through fMRI to argue that the limitation of this study is that activation changes were not measured by image acquisition before and after treatment (Jungblut et al., 2014). Cortese et al. (2015) found that in Italian MIT, all phonemic structure, speech automatism, prosody, communication, correct repetition, naming, and comprehension improved, while the adaptation of the MIT in the French language was developed by Belin et al. (1991).

### Case Studies

Because the case study method is more meticulous and concentrated, the examination and evaluation method of MRI plus scale is more common.

Van der Meulen et al. (2012) compared MIT interventions with those of two patients. After 6 weeks, patients with MIT improved 35 scores in repetition trained phrases, 50 scores in repetition, 7 scores in action naming, and 9 scores in comprehension (AAT). Seven scores were improved in Amsterdam Nijmegen Everyday Language Test (ANELT); 22.5 scores were improved in Sabadel Story Retell Task. Slavin and Fabus (2018) conducted a before-after MIT intervention in a 63-year-old man with chronic aphasia for 10 years. Unlike other studies, Slavin teamed up with a professional music therapist to intervene. The results found that MIT improved auditory comprehension skills, question answering, and repetition of BDAE after listening to the paragraphs. Breier et al. (2010) compared two patients with chronic aphasia with an average age of 53 and an average duration of 3.5 years. Using MR to observe hemisphere structural changes, patient 1 with MIT exhibited a significant increase in CIUs (>35%) after the first block of treatment. Patient 1 showed lateralization in the right hemisphere of MEG activity. Al-Janabi et al. (2014) used transcranial magnetic stimulation (rTMS) and MIT to intervene two aphasia patients with an average duration of 15 months and using MR to the before-and-after comparison. The results revealed that patients with MIT revealed significant activity increase in left BA44 and a decrease in right BA44. Patient 2 revealed significant activity increase in left BA44, right BA44, and left BA45. Tabei et al. (2016) used fMRI to observe a 48-year-old patient with a 3-year history of chronic aphasia before and after 9 days of intensive MIT. The results showed in fMRI that the patient had a significant activation of the medial frontal gyrus, inferior frontal gyrus, superior temporal gyrus, lentiform nucleus, and lingual gyrus of the right hemisphere.

### In the Research Using fMRI Measurement, the Main Concentrated Region of Interest in Brain

**Figure 2:**
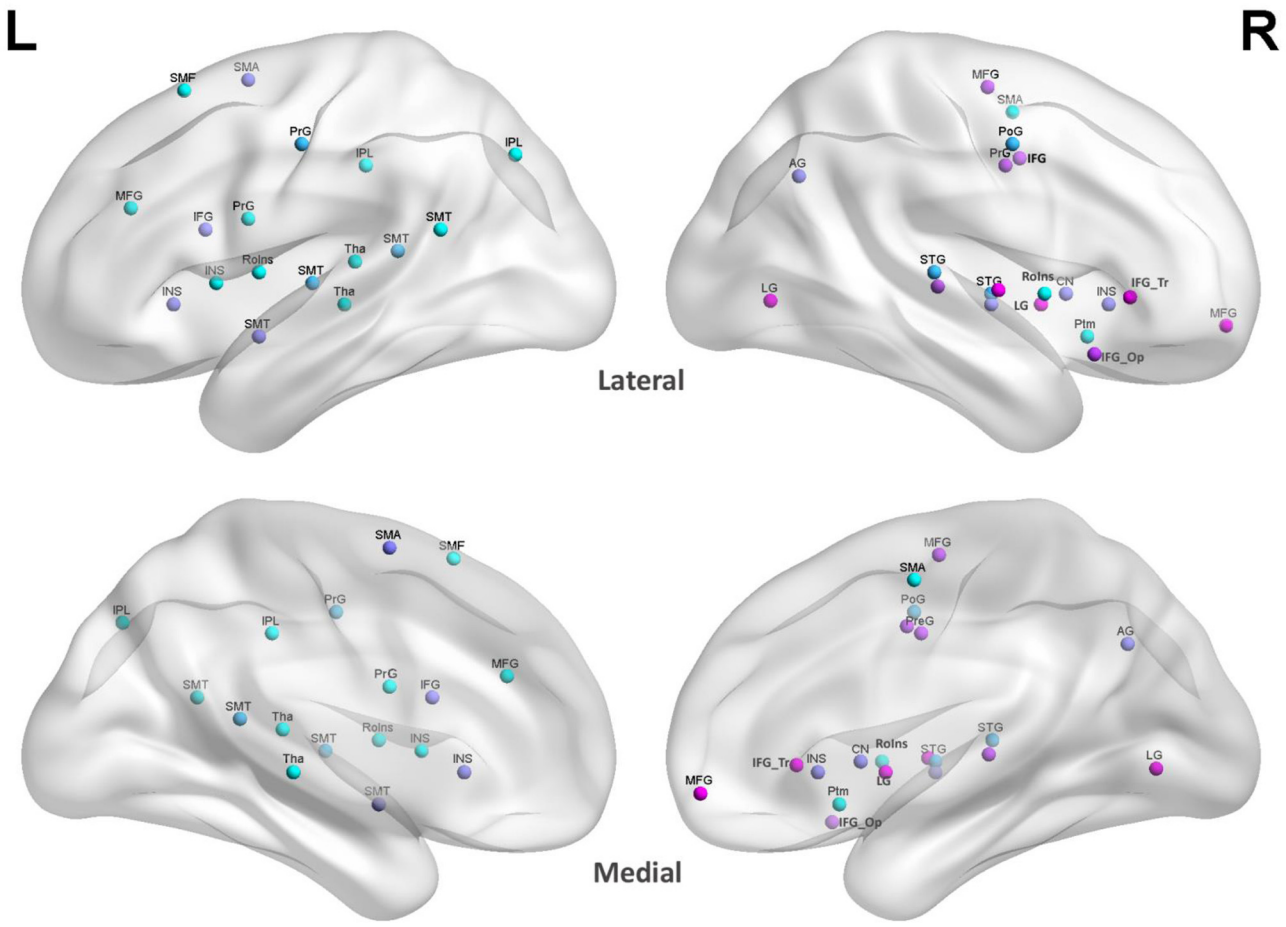
ROI in MIT-supported studies. The site colors were randomly generated to distinguish different ROI regions at the same site to prevent color overlap. L, left hemisphere; R, right hemisphere; Lateral, lateral cerebral hemisphere; Medial, medial cerebral hemisphere; SMT, superior and middle temporal gyrus; SMA, supplementary motor area; SMF, superior and middle frontal gyrus; PrG, pre-central gyrus; PoG, post-central gyrus; IPL, inferior parietal lobule; IFG, inferior frontal gyrus; INS, insula; MFG, middle frontal gyrus; RoIns, rolandic operculum insula; Tha, thalamus; LG, lingual gyrus; AG, angular gyrus; LN, lentiform nucleus; IFG_Tr, inferior frontal gyrus pars triangularis; IFG_Op, inferior frontal gyrus pars opercularis.

Through summarizing the studies in Table 1 which used fMRI to support MIT, we used the software BrainNet Viewer to locate the brain ROI (regions of interest). BrainNet Viewer is a brain network visualization tool for imaging connect omics. It can help researchers to visualize topological patterns of structural and to find functional brain networks derived from different imaging modalities (Xia et al., 2013). Using the BrainNet Viewer to locate the occurrence sites, it was found that all MIT-supported patients had more activation ROI in the right hemisphere than in the left hemisphere. The concentrated areas of ROI are the precentral gyrus, precentral sulcus, postcentral gyrus, middle frontal gyrus, superior temporal gyrus, superior temporal sulcus, middle temporal gyrus, inferior temporal sulcus, lingual gyrus, angular gyrus, etc. (Figure 2).

## Discussions

Our review selected 39 typical effective MIT experimental studies from 127 studies. Their common feature is the use of musical melody to intervene in aphasia, accompanied by effective evaluation. This analysis and discussion are based on the analysis of the intervention methods, evaluations, and effects of these studies.

### Feasibility Differences in Measurement Methods Between Clinical Trials With More Subjects and Case Studies

In these RCT studies with relatively more subjects, we found that objective imaging observation was not used as a primary means of effective monitoring. There may be some correlations to the therapeutic way of MIT. The one-to-one treatment and evaluation method will increase the working load of clinicians. If every participant is involved in the medical imaging test, the clinical workload, patient compliance, and financial support will all influence factors. Therefore, in more than 6 subjects of RCT studies in the past 10 years, only two articles with imaging observations were found. However, all of the RCT findings, including the two objective tests, confirmed the effectiveness of the subjective measurement scale of MIT. Because MIT requires individualized intervention and a long course of treatment, language assessment scales are the most convenient way of assessment. Compared with the high-cost evaluation of functional MRI, the scale evaluation of more than 15 patients with aphasia is easy to operate on and easy to compare before and after. In these MIT studies using MRI detection, the changes in cortical white matter and fiber bundles are apparent, which provide substantial evidence for the therapeutic effect of MIT and lay a foundation for the study of neural mechanisms.

However, due to the time-consuming, labor-intensive, and cost-intensive MRI examinations, most of these MIT's RCT studies have the following shortcomings: (i) there are some studies (Orellana et al., 2014) that could perform long-term MIT intervention experiments, and the imaging examinations are meticulous. Still, the number of samples is too small. Most of the samples comprised 6 participants; (ii) although there are 4 studies (Schlaug et al., 2009; Stahl et al., 2013; Zumbansen et al., 2014a; Cortese et al., 2015) that can match the minimum number of statistical subjects, there is no long-term intervention for comparison; therefore, the cumulative effect cannot be observed. The only one-time immediate effect is not enough to explain the mechanism. Therefore, in the future, how to ensure that both the demand for sample size and the long-term intervention of MRI detection can be achieved is matter of pressing academic concern.

### The Number of Musical Factors in the MIT Intervention Is Directly Linked to the Imaging Results

Previous literature has demonstrated several effective clinical results related to the recovery of musical melody-induced speech in the treatment of post-stroke aphasia. MIT (Albert et al., 1973), formally proposed by the American Academy of Neurology in 1973, is used to treat aphasia. In the early clinical treatment of non-fluency aphasia, Sparks et al. (1974) recorded the use of spectrum examples when training patients, with “Sprechgesang” as the core, requiring patients to follow the written melody. Zipse et al. (2012), Orellana et al. (2014), and Tabei et al. (2016), and others tend to use MIT treatment under more musical intervention, so their imaging results all show more features of active right hemisphere area. Moreover, although Zipse et al. (2012), Schlaug et al. (2014), Akanuma et al. (2015), and others used MIT recordings or provided MIT by general therapists, their intervention processes were all over 8 weeks. The natural melody factor in MIT makes the imaging results they obtained also reflected the active characteristics of the right hemisphere. Therefore, in the existing MIT experimental research, it is found that musical factors and the cumulative effect of time will directly affect the evidence that the right hemisphere of the brain participates in activities. Although rhythm is part of the music, as the rhythm is unpitched, we did not find a clear trend of activating the right hemisphere in the MIT intervention under the guidance of rhythm or language.

It is reported that the effects of the musical rhythm are observed in the left brain areas (Chen et al., 2008) and listening to musical rhythms recruits motor regions of the brain (Limb Charles et al., 2006; Limb et al., 2006; Thaut et al., 2014). However, these studies only focus on the music listening of healthy individuals or the rhythm perception of musicians. They are not the observation of MIT on patients with aphasia caused by stroke in the left hemisphere. Therefore, in the case of damage to the language center of the left hemisphere, patients treated with MIT can have correct oral speech output. This phenomenon confirms the mechanism of musical pitch from one side. But its brain mechanism still needs further study.

### The Neural Mechanism of MIT Based on Music

In the evidence summarized in previous MIT experimental studies, we found that the ROIs activated by MIT were the central anterior gyrus, central anterior sulcus, central posterior gyrus, middle frontal gyrus, superior temporal gyrus, superior temporal sulcus, middle temporal gyrus, inferior temporal gyrus, lingual gyrus, and angular gyrus of the right hemisphere. These areas include the frontal motor cortex (including Broca's area and ventral anterior motor cortex), which connects speech sensation and output, auditory cortex (including superior temporal gyrus and middle temporal gyrus), and parietal cortex (including angular gyrus and gyrus). MIT based on music activities, that is, MIT provided by professional music therapists, whether extracting lyrics from familiar songs or learning new fixed-pitch short melody for patients, affects the white matter structure of the auditory-motor neural circuit compensation to promote the ability to encode and integrate verbal information. This trans-hemisphere “mirror effect” has an important mechanism for the language recovery of patients with aphasia.

### Valuable Findings in Case Studies

It is found in literature retrieval that the evaluation methods of case studies are generally comprehensive and meticulous. Such qualitative studies reflecting the therapeutic effects of satisfactory MIT have more profound clinical implications for the brain regions it may activate. In the case reports retrieved in this paper, the evaluation criteria of early studies were generally international scales, mostly subjective scoring methods, and language recovery competence was based on scoring in different dimensions. In the recent 10 years of research, some medical imaging evidence of changes in brain structure at MIT to aphasia patients is easier to find in case reports (Schlaug et al., 2009; Al-Janabi et al., 2014; Tabei et al., 2016; Martzoukou et al., 2021). Besides, in the case study, whether the language assessment scale or fMRI was used, the subjective measurement and objective monitoring of patients have received sufficient concertation. Evidence of structural changes in patients' brain regions before and after also provides a factual neurological basis for MIT. It provides a realistic basis for the treatment of clinical aphasia.

### The Importance of Music Therapist at MIT

In the literature we reviewed, only 5 studies mentioned the participation of music therapists. Although MIT originated in speech therapy, MIT's guidance is melodic. It should be necessary for a correct rehabilitative approach by MIT to have specific training. For instance, the accuracy of melodic language needs a musical or music therapy formation. The rest of the literature does not mention the credentials of speech therapists and whether they have music learning experience. In fact, in MIT, treatment performed by music therapists includes instrumental accompaniment, melodic guidance, and songs inducement. Therefore, in the process of activating the vocabulary encoding of patients with aphasia, the instrument accompaniment, the professional, accurate melodic pitch, and the guidance from music therapists to play and sing are all combined to activate the melodic “lyrics” of the episodic memory network and promote the output of spoken language.

### Expectations for Future MIT Development

Through MIT's RCT studies, the left and right brains were found to have different processing advantages. The functional areas responsible for music melody processing and memory retrieval are more concentrated in the auditory cortex of the right brain temporal lobe. Therefore, it is speculated that the left brain is more responsible for language functions. After damage sustained by the dominant hemisphere, MIT may activate the auditory cortex corresponding to music processing in the right brain and activate the right brain language motor area corresponding to the Broca's area of the left brain through the conduction of the right arcuate track to achieve compensation and guide the patient's language output, to achieve the purpose of language communication (Merrett et al., 2014). However, 90% of the literature we reviewed so far was RCT studies on Western language aphasia; only 10% of the literature comes from East Asian language aphasia (Japanese and Korean), while the MIT intervention in Chinese Mandarin aphasia trials has not been found in internationally registered clinical trials. Compared with Western languages, East Asian languages, as a tonal language (including four or more tones), have a more bilateral distribution of brain nerve circuits than Western languages represented by English (Liang and Du, 2018). However, despite this, the neural mechanism of the effect of MIT on East Asian languages has not been verified by a large sample of experiments.

It should be noted that, according to the high incidence of aphasia. However, relatively effective treatment methods were developed. A large amount of imaging evidence has not supported MIT, nor has it been endorsed by large cohort studies. This may be due to factors such as MIT's over-reliance on therapists, its unitary approach, lack of computerization, and individual patient differences. From existing evidence, MIT is effective and has positive results of scale testing. In the future, researchers should try the use of technology to develop music artificial intelligence evaluation and training tools, streamline and step the operation of MIT, reduce the human cost, and, on this basis, cooperate with imaging detection, and then conduct large sample experiments, so that the clinical and scientific value of MIT will be maximized in the future.

## Data Availability Statement

The original contributions presented in the study are included in the article/supplementary material, further inquiries can be directed to the corresponding author/s.

## Author Contributions

All authors listed have made a substantial, direct, and intellectual contribution to the work and approved it for publication.

## Funding

This research received a grant from Scientific Research Project of Establishment of the Winter Olympics Sports Injury Rehabilitation Diagnosis and Treatment System and Green Channel Demonstration, No. 2018YFF0301104 (to JL).

## Conflict of Interest

The authors declare that the research was conducted in the absence of any commercial or financial relationships that could be construed as a potential conflict of interest.

## Publisher's Note

All claims expressed in this article are solely those of the authors and do not necessarily represent those of their affiliated organizations, or those of the publisher, the editors and the reviewers. Any product that may be evaluated in this article, or claim that may be made by its manufacturer, is not guaranteed or endorsed by the publisher.
